# Chronic Proton-Pump Inhibitor Therapy and Fracture Risk in Women Aged Between 50 and 65 years: A Retrospective Case-Control Study

**DOI:** 10.7759/cureus.28429

**Published:** 2022-08-26

**Authors:** Nimesh Patel, Mohamed Fayed, Priyansh Faldu, Wissam Maroun, Janki Chandarana

**Affiliations:** 1 Anesthesiology, Pain Management and Perioperative Medicine, Henry Ford Health, Detroit, USA; 2 Medicine, BJ (Byramjee Jeejeebhoy) Medical College Ahmedabad, Ahmedabad, IND; 3 Family Medicine, Beaumont Health, South Gate, USA

**Keywords:** osteoporosis, chronic smoking, body mass index (bmi), fall injury, simple fall, senile osteoporosis, proton-pump inhibitor, pathological femoral shaft fracture, prevention of osteoporosis, osteoporosis management

## Abstract

Introduction

Chronic proton-pump inhibitor (PPI) prescription is on the rise in the last decade with an increased prevalence in the elderly population. For most patients, this class of drugs is the primary treatment for various diseases. Even though PPIs are generally safe, long-term use has been associated with multiple adverse effects like bone fractures. The extent of the association between PPI and fracture is still unclear in women aged between 50 and 65 years. Besides, many other variables and risk factors must be accounted for in the analysis of this relation.

Methods

This is a retrospective case-control study looking at women 50-65 years of age who presented to Genesys Health for a low-impact fall. Data were extracted from electronic medical records and fracture outcomes; PPI therapy exposure and duration were determined. Chi-square analysis was performed to determine the association between chronic PPI therapy and fracture outcome and independently analyzed for major risk factors of osteoporosis, including smoking, low body mass index, and cancer.

Results

Patients in the chronic PPI therapy group were found to have a decreased fracture outcome overall in each subcategory of risk factors. When adjusting for all risk factors, there was a significant but weak association between chronic PPI therapy and increased fracture outcome.

Conclusion

With different results from previous studies, this study sheds new light on this debate. More studies need to be carried out to determine the association between chronic PPI therapy and fracture outcomes in postmenopausal women.

## Introduction

Chronic proton-pump inhibitor (PPI) prescription is on the rise in the last decade with an increased prevalence in the elderly population [1-3]. For most patients, this class of drugs is the primary treatment for various diseases, including peptic ulcer disease, esophagitis, prevention of nonsteroidal anti-inflammatory drugs associated with ulcers, gastroesophageal reflux disease, Zollinger-Ellison syndrome, and functional dyspepsia [4]. Even though PPIs are generally safe, long-term use has been associated with multiple adverse effects like bone fractures, hypomagnesemia, microscopic colitis, kidney disease, community-acquired pneumonia, and increased risk of *Clostridium difficile* infection [5]. Furthermore, PPIs are overused in outpatient and inpatient settings, especially in intensive care units [6]. As a result, this worldwide and often open-ended use of PPIs can have negative and unintended consequences in the long run.

Several studies have evaluated the association between PPI usage and fracture risk [7-14]. Furthermore, PPI use has been associated with osteoporosis [15-17]. This led to speculations about the increased fracture risk in postmenopausal women and has been observed in multiple studies [18-19]. Nonetheless, some studies showed that PPI use in postmenopausal women was not associated with an increased risk of hip fracture but modestly associated with other fractures [20]. Also, some studies have shown that PPI use was not related to fractures in patients aged 50-79 without risk factors [21].

To date, no prospective, randomized, blinded controlled trials have been done to analyze the association between chronic PPI therapy and fracture risk. The extent of the association between PPI and fracture is still unclear in women aged between 50 and 65 years. Besides, many variables and risk factors must be accounted for in the analysis of this relation. Our study was conducted to determine if there is a clinically significant increased outcome in the osteoporotic fracture in women 50-65 years on chronic PPI therapy.

## Materials and methods

This was a retrospective, case-control study that included women aged between 50 and 65 years presenting to Genesys Health for a low-impact fall. We received approval from the institutional review board, and the approval number was 11564273; the board's name is Genesys Health. A low-impact fall was defined as a fall from no greater than standing height due to slipping, tripping, or stumbling on the same level. This was determined by the International Classification of Diseases (ICD)-9 and ICD-10 codes. High-impact falls, defined as falls resulting from a sporting injury or falling from a height greater than standing, were excluded. If ICD-9/ICD-10 codes did not capture the type of impact, it was determined by the patient's chart review (history section or emergency department provider's note). Patients who were selected must have had more than one prior admission/visit for any reason to determine exposure and duration of PPI use. Those with only one admission/visit were excluded from the study.

Fracture outcome was determined by reviewing each patient's chart at the fall visit/admission, and the acute fracture was determined from the imaging section. Any mention of an old fracture was excluded. Chronicity of PPI therapy, defined as more than one year of use, was determined by prescription entry dates from the patient's outpatient medication list. Any PPI script that was ordered in the inpatient setting was excluded. Risk factors for osteoporosis (smoking, low body mass index (BMI) <18 kg/m^2^, Crohn's disease, ulcerative colitis, hyperparathyroidism, chronic kidney disease, cancer, and rheumatoid arthritis) were flagged. Risk factors that could alter fracture risks, such as medications including bisphosphonates and thiazide, were also flagged. Each risk factor was independently analyzed for fracture outcome with chronic PPI therapy compared to no PPI therapy. Categorical variables were summarized with frequencies and proportions and were compared using the Chi-square test or Fisher's exact test. A p-value <0.05 was considered statistically significant.

## Results

**Figure 1:**
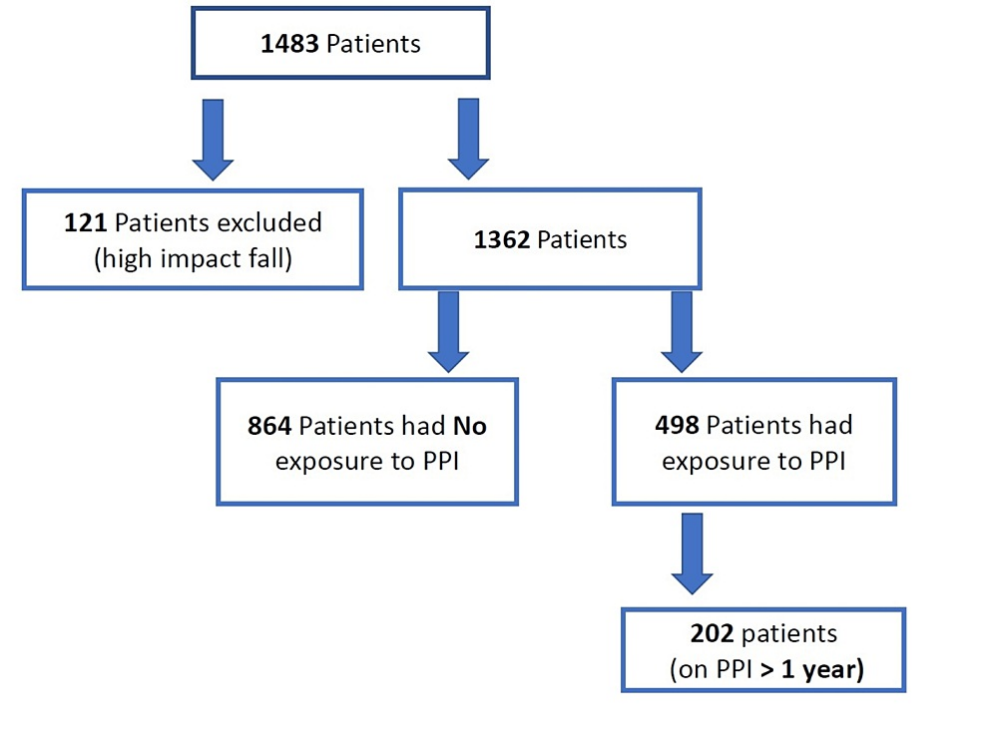
Total number of patients recruited. PPI: proton-pump inhibitor.

A total of 1,483 patients met the inclusion criteria. One hundred twenty-one patients were excluded due to having high-impact falls. Of the remaining 1,362, 864 patients had no exposure to PPI therapy, and 498 patients had PPI exposure. Only 202 patients had exposure to PPIs for greater than one year. In total, 864 patients (81.1%) were identified not to have been exposed to PPI, while only 202 patients (18.9%) were determined to have been on PPIs greater than one year (Figure 1).

**Figure 2:**
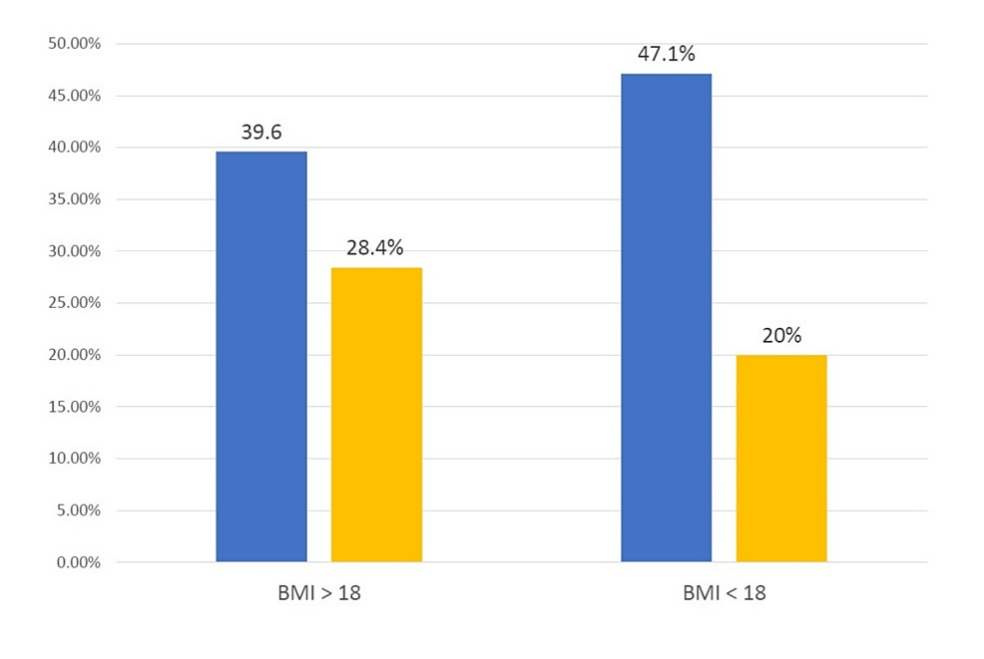
Fracture outcomes in patients with regular and low BMI. Blue column indicates no PPI,  and yellow column indicates PPI for more than one year. BMI: body mass index; PPI: proton-pump inhibitor.

Overall fracture risk was decreased by about 10% in patients on long-term PPI therapy (28.2%) as opposed to patients with no PPI use (39.7%), and this was clinically significant (X^2 ^= 9.2, p = 0.002); odds ratio is 0.6 (95% confidence interval: 0.43 to 0.83) (p-value = 0.002). Yet, these data were not adjusted for major risk factors of osteoporosis. Each category of major risk factors was independently analyzed. The data were not included for those risk factors with a very small sample size due to invalid clinical significance. Regarding the risk category of low BMI (<18 kg/m^2^), only 22 patients (3%) were identified to have been on long-term PPI therapy. The fracture risk was reduced in patients with regular BMI on long-term PPI therapy. Still, the decrease was more pronounced in patients with a low BMI (X^2 ^= 8.444, p = 0.004) (Figure 2).

**Figure 3:**
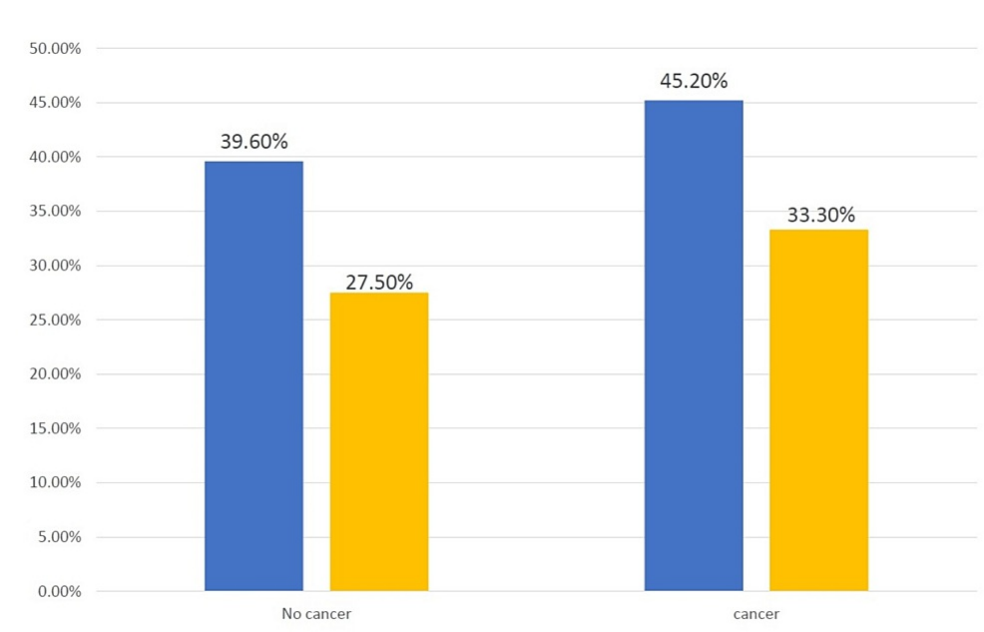
Fractures outcome in patients with cancer. Blue column indicates no PPI, and yellow column indicates PPI for more than one year. PPI: proton-pump inhibitor.

Concerning the cancer risk category, only 55 patients (6%) were identified to have been on long-term PPI therapy. The fracture risk was low in those patients on long-term PPI therapy without cancer. Still, the decrease was more pronounced in cancer patients (X^2 ^= 8. 973, p = 0.003) (Figure 3).

**Figure 4:**
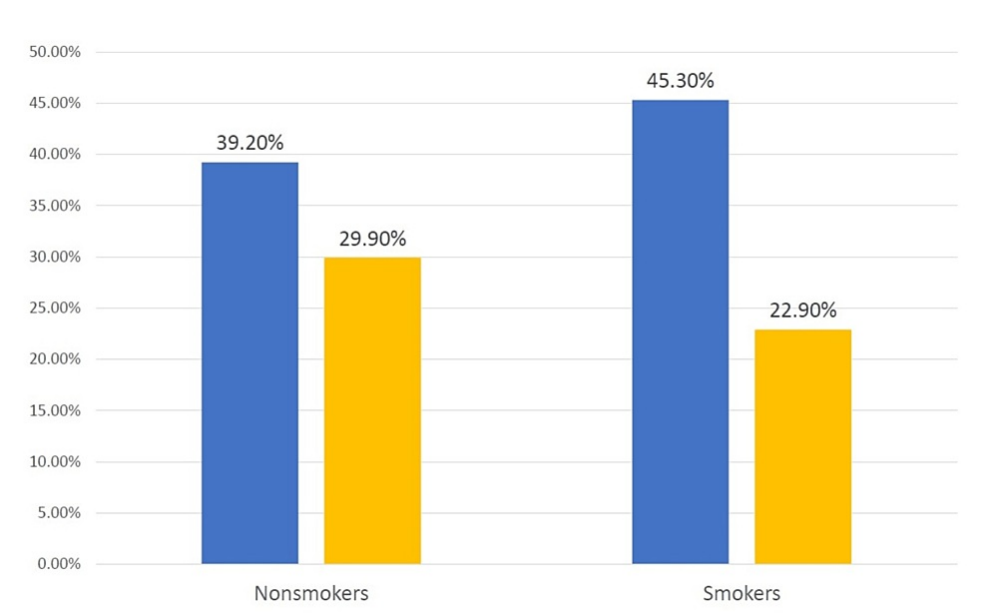
Fractures outcome in smokers. Blue column indicates no PPI, and yellow column indicates PPI for more than one year. PPI: proton-pump inhibitor.

Concerning smoking, about 123 patients (12%) were identified to have been on long-term PPI therapy. The fracture risk was low in nonsmoker patients on long-term PPI therapy. Yet, the decreased fracture risk was more pronounced in smokers (X^2 ^= 4.741, p = 0.029) (Figure 4).

All three separate analyses were clinically significant. Nonetheless, adjusting for the variables, such as cancer, ulcerative colitis, low BMI, smoking, and thiazides, there was a significant but weak association between the PPI more than one-year group and fracture outcome (R² = 0.003, p = 0.002).

## Discussion

Osteoporosis is a multifactorial skeletal disease characterized by reduced bone strength and increased susceptibility to bone fractures [22-23]. Postmenopausal women are far more likely to be affected by osteoporosis and associated fractures than men [24]. According to an estimate by the National Osteoporosis Foundation (NOF), it is estimated that there are 9.1 million women who have osteoporosis. Because of osteoporosis, a 60-year-old woman has almost double the lifetime risk of fracture than a man of the same age [25]. The spine, hip, distal forearm, and proximal humerus are some of the common sites for osteoporotic fractures. Thus, osteoporotic fractures significantly affect the quality of life and cause significant morbidity among postmenopausal women [26-27]. Vertebral fractures are the most common and are associated with functional limitation due to acute back pain. While most cases resolve, some may progress to chronic back pain and disability [28-29]. Hip fractures are also associated with acute pain and loss of functionality. The recovery rate is slow, and most patients require hospitalization. While distal radial fractures have positive recovery outcomes, they also lead to acute pain and loss of function in most cases [26-27]. Chronic PPI use has been associated with osteoporosis [15-17]. Mechanisms suggested include hypergastrinemia, hypochlorhydria, and possible direct bone effects [18]. Hence, it is speculated that chronic PPI by increasing osteoporosis risk might increase fracture risk in postmenopausal women. As discussed earlier, this might lead to chronic pain, disability, and loss of function.

Our study results show a significantly reduced fracture risk among those on PPI for more than one year compared to those not on PPI. In addition, the risk of fracture decreased in patients on chronic PPI even when stratified by major risk factors. This conclusion was not consistent with the conclusions seen in most studies done on postmenopausal women [19]. A possible explanation behind the inconsistency with previous results might include sample distribution in each group: As mentioned in the results, only about 18.9% were identified to have been on chronic PPI as opposed to about 81.1% not on PPI therapy. Furthermore, with the evaluation of each risk category, the reduced sample size of those patients on chronic PPI therapy was more significant. Access to data from multiple hospitals or a larger population might have increased the sample size and the study's power. Another reason that may have contributed to our results compared to studies done in the past is that our patient population was only in the inpatient or the emergency room setting. This suggests our patients probably have more comorbidity than patients presenting for a low-impact fall in an outpatient setting such as the clinic or urgent care. Also, the difference in comorbid conditions between groups was not determined. One group may have had a higher number of patients with the comorbid disease, increasing the number of falls or risk factors for osteoporosis, resulting in a higher chance of fracture outcome.

Moreover, patients included were from one hospital and likely residing in the Grand Blanc region. This could affect fracture risk due to the prevalence of certain diseases or lack of sun exposure that could affect vitamin D levels and alter bone density. Besides, our data did not analyze the percentage of fracture outcomes within each racial category. In addition, biases are a common limitation in this study by design. The patient's home medication list was self-reported, which might be subject to recall bias. Also, patients may have used an over-the-counter PPI intermittently, so this information may have been underreported. Likewise, patients' compliance with PPIs may be variable, which was not further assessed. Hence, cross-referencing the outpatient medication list to an external outpatient data set would have contributed to minimizing recall bias. Another limitation of this study was not categorizing the major osteoporotic sites known to affect a patient's overall functionality, as discussed earlier. Determining fracture outcomes at major osteoporotic sites such as the hip, spine, and distal forearm would have been beneficial. Also, reporting different doses/strengths of PPIs would have been beneficial in this study as it would have determined if PPI use has a dose-dependent effect on fracture outcome.

However, when adjusting for some of the major risk factors for osteoporosis, such as cancer, ulcerative colitis, low BMI, smoking, and medications, such as thiazides, that could alter fracture outcome, there is a significant but weak association between the PPI more than one-year group and fracture outcome. This finding was consistent with the conclusions seen in most previous studies. Practice guidelines suggest lowering the PPI dose to the lowest effective dose to treat symptoms or disease or switching to less potent medications such as H2 blockers to minimize the risk of adverse effects and complications. Providers should continue practicing this recommendation. With opposing results, this study sheds new light on this debate. More studies need to be done to determine the association between chronic PPI therapy and fracture outcomes in postmenopausal women.

## Conclusions

Our study showed that the chronic use of PPI over one year is associated with a low fracture probability. With different results from previous studies, this study sheds new light on this debate, and more studies need to be done to determine the association between chronic PPI therapy and fracture outcomes in postmenopausal women, especially in regions with various influencing factors including sun exposure, racial differences, or access to health care.
